# Lung Cancer Mortality in Tuscany from 1971 to 2010 and Its Connections with Silicosis: A Space-Cohort Analysis Based on Shared Models

**DOI:** 10.1155/2018/4964569

**Published:** 2018-01-28

**Authors:** Emanuela Dreassi

**Affiliations:** Dipartimento di Statistica, Informatica, Applicazioni “G. Parenti”, Università di Firenze, Viale Morgagni 59, 50134 Firenze, Italy

## Abstract

Lung cancer mortality in Tuscany (Italy) for males, from 1971 and 2010, is investigated. A hierarchical Bayesian model for space-time disease mapping is introduced. Such a model belongs to the class of shared random effect models and exploits the birth-cohort as the relevant time dimension. It allows for highlighting common and specific patterns of risk for each birth-cohort. The results show that different birth-cohorts exhibit quite different spatial patterns, even if the socioeconomic status is taken into account. In fact, there were different occupational exposures before and after the Second World War. The birth-cohort 1930–35 exhibits high relative risks related to particular areas. This fact could be connected with occupational exposure to risk factors for silicosis, perhaps a prognostic status for lung cancer.

## 1. Introduction

Descriptive epidemiology focuses on the variation of disease occurrence among populations. Studies of time-space variations in mortality are widely used in etiologic research in order to evaluate environmental exposure. Models that jointly describe space and time variation of disease risk have been extensively proposed. All of them are essentially extensions of the hierarchical Bayesian model by Besag et al. [1]. See, for example, Bernardinelli et al. [2], Waller et al. [3], Assunção et al. [4], Sun et al. [5], and Knorr-Held and Besag [6].

In particular, Knorr-Held [7] suggests a generalized linear mixed model where space, time, and different specifications of interaction terms between space and time effects are involved. Following the latter, Lagazio et al. [8] proposed the same model where the birth-cohort is the main time dimension.

Taking birth-cohort as time dimension is motivated by several biological reasons, which make it preferable to calendar period when considering the consequences of a change in the prevalence of human exposure to risk factors in the population, even if it requires the mortality or incidence data for a long time span. As a rule, age effects are related to the natural history of the disease, while birth-cohort and/or calendar period effects depend on different temporal patterns of prevalence of human exposure to risk factors. Therefore, an appropriate selection of the time scales yields important clues about the biology of the disease, and it is fundamental to correctly understand the evolution of the epidemics.

Lung cancer is the most common type of cancer in the world since 1985 and its spatiotemporal variation is examined by an extensive epidemiological literature. See, for example, Boyle and Ferlay [9] and Bray and Weiderpass [10]. The adopted time scale is the calendar period (e.g., [11]) or birth-cohort [12, 13].

As regards mortality for lung cancer, socioeconomic factors are assumed to be associated with individual exposure to risk factors (e.g., tobacco smoking or occupational exposure). In this case, the biology of the process of carcinogenesis suggests that more than 10–15 years (the latency time) should run between exposure and mortality (see [14]). See the foundational papers by Glick [15] and Mayer [16] for a discussion and a motivation of spatial analysis of cancer mortality as well as for the theoretical models that take into account the process of carcinogenesis. A strong connection between smoking habits and material deprivation has been stressed by several studies (see, e.g., [17]). Material deprivation indicators usually refer to the occurrence of subject states such as unemployment, low education, living in a very small dwelling, overcrowding, and not having a car (e.g., see [18–22]). So far, material deprivation indicators have been used as aggregate-level covariates to adjust ecological regression coefficients in small area studies [23]. In fact, a strong association with area-based deprivation and mortality was repeatedly found on one side and area-based deprivation and exposure to environmental/individual hazards on the other [24–26]. Relevant risk factors could be considered in the model with the inclusion of a material deprivation index (see [27–30]).

In this paper, a space-cohort analysis for disease mapping is developed. A model, more parsimonious than Lagazio et al. [8], is introduced in Section 2. Such a model, called Shared Model for Birth Cohort (SMBC) in the sequel, is actually a simple version of the usual intrinsic conditional autoregressive (CAR) models, but it shows reasonably good performance when comparing a disease among the strata of a given population. So far, shared models have been used for various reasons, such as to compare two or more diseases [31–33], to compare the same disease for two genders in space and time [34, 35], and for ecological regression analysis [36]. Using shared models to compare the same disease between two different birth-cohorts, as done by SMBC, seems to be new.

To assess its behavior, SMBC is then applied to a case study. The data, described in Section 3, are the lung cancer death certificates collected for males residing in the 287 municipalities of the Tuscany Region (Italy) from 1971 to 2010. The information on material deprivation is based on census data collected from the Italian Statistical Institute (ISTAT) in the years 1961, 1971, 1981, and 1991. Four variables are used as indicator of socioeconomic inequalities, namely, unemployment, low education (less than 6 years of schooling), being a tenant, and the absence of a bathroom in the house.

Finally, the results obtained by applying SMBC to the above data are discussed in Section 4. Overall, the performances of SMBC look encouraging and endorse some etiologic hypotheses of mortality for lung cancer highlighted by the medical literature. We refer to Section 4 for a detailed discussion. Here, we just note that occupational exposure to risk factors for silicosis, perhaps a prognostic status for lung cancer, is strongly supported by our application of SMBC.

## 2. Space-Cohort Models

Let *y*_*it*_ denote the observed number of deaths for the *i*th area and the *t*th cohort, with *i* = 1,…, *I* and *t* = 1,…, *T*. In the sequel, following Besag et al. [1], each *y*_*it*_ is assumed to have a Poisson distribution with mean value *θ*_*it*_*E*_*it*_, where *E*_*it*_ is the number of expected cases and *θ*_*it*_ the relative risk. Also, the random variables *y*_*it*_ are conditionally independent given the matrix {*θ*_*it*_:  *i* = 1,…, *I*; *t* = 1,…, *T*}.

### 2.1. Space-Time Interaction Models

In Lagazio et al. [8], *θ*_*it*_ is modelled as

(1) $$\begin{array}{c} \log\left(\;\theta_{it}\;\right)=\beta_{0}+\beta_{1}x_{it}+v_{i}+u_{i}+\phi_{t}+\psi_{it}, \end{array}$$

where *x*_*it*_ is a time-dependent covariate, *v*_*i*_ and *u*_*i*_ are, respectively, the unstructured and structured spatial variability terms [1], *ϕ*_*t*_ is an unstructured effect for the *t*th cohort, and *ψ*_*it*_ is an area specific cohort effect (the interaction term spatially structured). The prior distributions for *v*_*i*_, *u*_*i*_, and *ϕ*_*t*_ are multivariate normal with mean zero and precision matrices *τ*_*v*_**K**_*v*_, *τ*_*u*_**K**_*u*_, and *τ*_*ϕ*_**K**_*ϕ*_, respectively. Proper Gamma priors with very high dispersion have been assumed for the hyperparameters *τ*_*v*_, *τ*_*u*_, *τ*_*ϕ*_.

The structure matrices [37] **K**_*v*_, **K**_*u*_, and **K**_*ϕ*_ are specified following the different nature of effects. In particular, **K**_*v*_ and **K**_*ϕ*_ are taken to be identity matrices, while the structured spatial component **K**_*u*_ is modelled as an intrinsic Gaussian autoregression [1, 2, 37]. Precisely, the entries *k*_*ij*_ of **K**_*u*_ are given by

(2) $$\begin{array}{c} k_{ij}=\left\{\begin{array}{cc} -1 & \text{if areas }i\text{ and }j\text{ are contiguous}\\ 0 & \text{if areas }i\text{ and }j\text{ are not contiguous}\\ n_{i} & \text{if }i=j, \end{array}\right. \end{array}$$

where *n*_*i*_ is the number of areas contiguous to the *i*th one.

With this definition of **K**_*u*_, the mean of the conditional distribution of *u*_*i*_ given all the other *u* terms is 1/*n*_*i*_∑_*i*~*j*_*u*_*j*_, where *i* ~ *j* indicates that areas *i* and *j* are spatially contiguous. The conditional precision is given by *n*_*i*_*τ*_*u*_.

Interaction terms are assumed to be structured in space. The prior distribution is multivariate normal with mean zero and precision matrix *τ*_*ψ*_**K**_*ψ*_. Again, a highly dispersed proper Gamma prior was used for *τ*_*ψ*_. The structure matrix **K**_*ψ*_ was defined as the Kroneker product between the matrices **K**_*u*_ and an identity matrix [7, 37].

The precision is *n*_*i*_*τ*_*ψ*_ for *t* = 1 or *t* = *T* and 2*n*_*i*_*τ*_*ψ*_ for *t* = 2,…, *T* − 1.

To complete the model specification, highly dispersed normal prior has been assumed for the regression coefficients *β*_0_ and *β*_1_.

Posterior distributions of the parameters of interest have been approximated using Gibbs sampling. After a burn-in of 100,000 iterations, we retained 1,000 samples taken from the last 100,000 iterations. The posterior distributions have been summarised using the posterior mean. Because of the high number of terms in the model, convergence has been assessed only on a subset of the identifiable parameters. Gelman and Rubin [38] test and partial autocorrelation plots have been used to check for achieved convergence of relative risks and of the *τ* hyperparameters.

The interaction terms represent the differences of the spatial pattern between two or more birth-cohorts.

### 2.2. Shared Models for Birth-Cohort Analysis

As noted in Section 1, in this paper, shared models are used for a space-time (birth-cohort) analysis of relative risk and the resulting model is denoted by SMBC.

Shared models allow highlighting the differences between the spatial patterns of two or more birth-cohorts and represent an effective alternative to space-time interaction models. In fact, usually, shared models provide reliable information and lead to more parsimonious models.

In SMBC, following Besag et al. [1] and Knorr-Held and Best [31], it is assumed that

(3) $$\begin{array}{c} \log\left(\;\theta_{it}\;\right)=\beta_{0}+\beta_{1}x_{it}+\alpha_{t}+\delta_{it}, \end{array}$$

where *α*_*t*_ is a birth-cohort specific intercept (overall risk level) and *δ*_*it*_ a spatial term. In turn, *δ*_*it*_ is decomposed into a shared and birth-cohort specific spatial effect; namely,

(4) $$\begin{array}{c} \delta_{it}=\omega_{t}\psi_{i}^{\left(s\right)}+\psi_{it}^{\left(p\right)}=\omega_{t}\left(\;\gamma_{i}^{\left(s\right)}+\phi_{i}^{\left(s\right)}\;\right)+\left(\;\gamma_{it}^{\left(p\right)}+\phi_{it}^{\left(p\right)}\;\right), \end{array}$$

where

- the shared component *ψ*_*i*_^(*s*)^ is the convolution of an unstructured spatial effect *γ*_*i*_^(*s*)^ and a spatially structured term *ϕ*_*i*_^(*s*)^;
- the specific component *ψ*_*it*_^(*p*)^ is the convolution of an unstructured spatial effect *γ*_*it*_^(*p*)^ and a spatially structured term *ϕ*_*it*_^(*p*)^;
- the scale parameter *ω*_*t*_ allows the shared component to vary per birth-cohort by a constant factor.

The prior distributions are as follows. The intercept *α*_*t*_ has a flat “noninformative” prior (a centered normal distribution with “large” variance). The terms log⁡*ω*_1_,…, log⁡*ω*_*T*_, constrained to ∑_*t*=1_^*T*^log⁡*ω*_*t*_ = 0, are jointly normal with zero mean and covariance matrix

(5) $$\begin{array}{c} \Sigma_{\omega }=\sigma_{\omega }^{2}\left(\begin{array}{cccc} 1 & -\frac{1}{\left(T-1\right)} & \cdots & -\frac{1}{\left(T-1\right)}\\ -\frac{1}{\left(T-1\right)} & 1 & \cdots & -\frac{1}{\left(T-1\right)}\\ \vdots & \vdots & \vdots & \vdots \\ -\frac{1}{\left(T-1\right)} & -\frac{1}{\left(T-1\right)} & \cdots & 1 \end{array}\right). \end{array}$$

The heterogeneity terms *γ*_*i*_^(*s*)^ and *γ*_*it*_^(*p*)^ are independent centered normal with precision parameters  *λ*_*γ*_ and *λ*_*γ*_*t*__. In order to cope with the spatial structure, the random effects *ϕ*_*i*_^(*s*)^, *ϕ*_*it*_^(*p*)^ are modelled as intrinsic CAR models, as discussed, for example, in Lee [39], with precision parameters *λ*_*ϕ*_ and *λ*_*ϕ*_*t*__.

Hyperprior distributions for the precision parameters *λ*_*γ*_, *λ*_*γ*_*t*__, *λ*_*ϕ*_, and *λ*_*ϕ*_*t*__ are assumed to be Gamma (0.5,0.0005).

Posterior distributions of the parameters of interest have been sampled by Gibbs sampling.

Once SMBC is implemented, an inspection of the estimated values of *ψ*_*i*_^(*s*)^ and *ψ*_*it*_^(*p*)^ provides valuable information about common and uncommon risk factors for different birth-cohorts. This procedure is exemplified in Section 4, where SMBC is applied to a real situation.

## 3. Data

Lung cancer death certificates were collected for males residing in the 287 municipalities of the Tuscany Region (Italy) from 1971 to 2010. For the 40 years analyzed, amounting to a total of 34,666,951 person-years, the number of recorded death certificates is 66856.

The data were made available by the Tuscany Regional Government under the research project* Tuscany Atlas of Mortality 1971–1994* (see [40]) and by the Regional Mortality Register for the period 1995–2010. Deaths and corresponding populations for each municipality were cross-classified in 10 age-classes (40–45,45–50,…, 80–85,85 and over; the first eight age-classes being omitted) and 8 calendar periods (1971–74,1975–79,1980–84,…, 2005–10).

The 1971–2010 database of lung cancer mortality, divided by gender at a municipality level, allows us to investigate several birth-cohorts.

**Figure 1:**
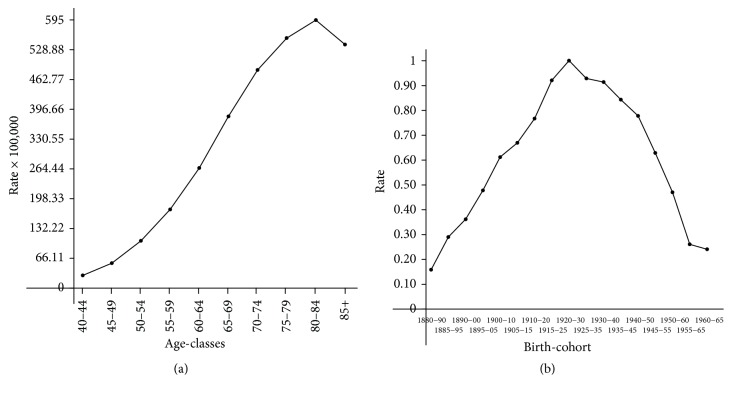
Epidemic curves for age (a) and cohort (b) dimensions.

The expected number of cases for each municipality was evaluated by the predicted age-specific reference rates calculated by the age-cohort model (see [41]) for the whole Tuscany Region.  Figure 1 shows the epidemic curve for the considered age-classes (a) and birth-cohorts (b). The reference birth-cohort is 1920–30, when the epidemic reached its maximum. Presumably, the exposure to smoking for such a cohort began in the 1940s, after the Second World War. This model allows disentangling age effects from birth-cohort components present in longitudinal mortality data. In this way, the expected number of cases is adjusted by age and is not affected by cohort effects.

Observed and expected cases were subsequently aggregated along the diagonals of the Lexis diagram representing birth-cohorts, thus collapsing on the age dimension. For the space-time analysis, we focused on the six birth-cohorts (1905–15,…, 1930–40).  Table 1 illustrates incidence rates and observed cases for the whole Tuscany Region, as well as the correspondence among birth-cohorts, age-classes, and calendar periods.

Data on material deprivation were taken from census data collected from Italian Statistical Institute (ISTAT) in the years 1961, 1971, 1981, and 1991. Four variables were used as indicator of socioeconomic inequalities: unemployment, low education (less than 6 years of schooling), being a tenant, and the absence of a bathroom in the house. A composite index was then computed as the sum of the *z*-score of the proportion of people with the conditions listed above for each municipality (see [28], for details).

The space-time dynamics of the association between mortality and deprivation level were studied using a deprivation score which results in a meaningful absolute level across the years. We considered socioeconomic factors observed in 1961, 1971, 1981, and 1991 and imputed, using a first-order random walk process, in 1956, 1966, 1976, 1986, and 1996. Further, we considered a temporal lag of 10 years between these and mortality.

## 4. Results

To compare birth-cohorts 1905–15 and 1930–40, we describe estimates for space-time interaction terms from the Lagazio et al. [8] model and the specific terms from the proposed model (SMBC). We focused on these birth-cohorts since their comparison seemed to be the most meaningful. In fact, the comparison highlights differences before and after Second World War. In addition, 1905–15 and 1930–40 are the youngest and oldest cohorts for which age-classes between 60 and 75 years were observed. Consequently, such cohorts have a substantial observed number of events.

**Figure 2:**
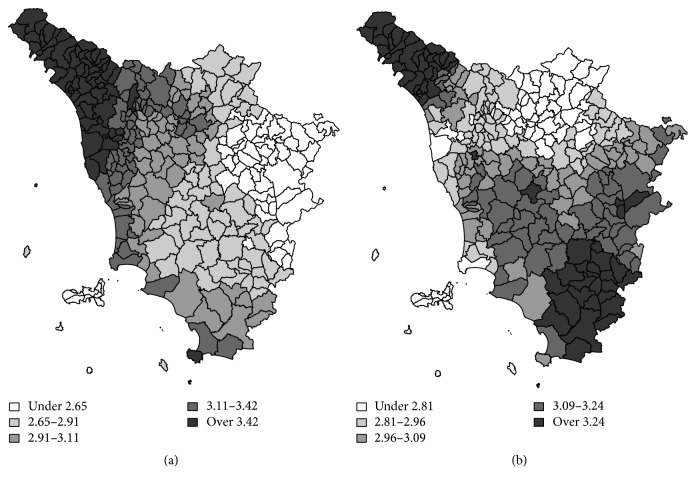
Maps of the interaction terms from the Lagazio et al. 2001 model [8]: birth-cohorts 1905–15 (a) and 1930–40 (b).

Figure 2 shows the estimates for the space-time interaction terms. The areas with the highest mortality (north-western part of the region) exhibit a decrease in relative risk, while the low-risk areas in the south-eastern part of the region behave in the opposite way, still showing an increase in relative risk.

**Figure 3:**
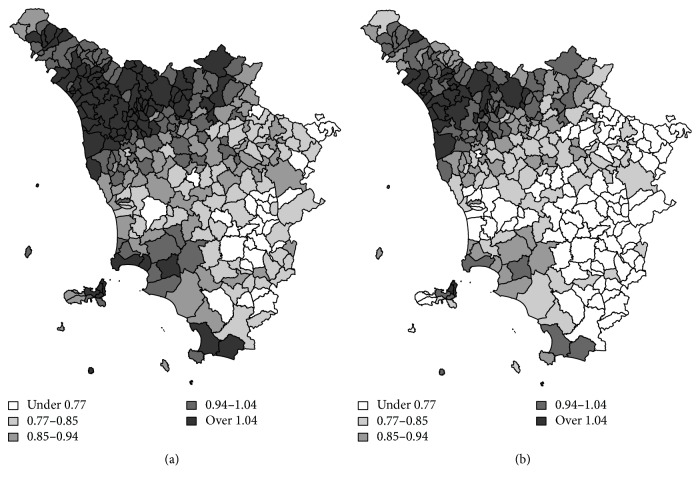
Relative risks for lung cancer mortality from SMBC model: for birth-cohort 1905–15 (a) and birth-cohort 1930–40 (b).

**Figure 4:**
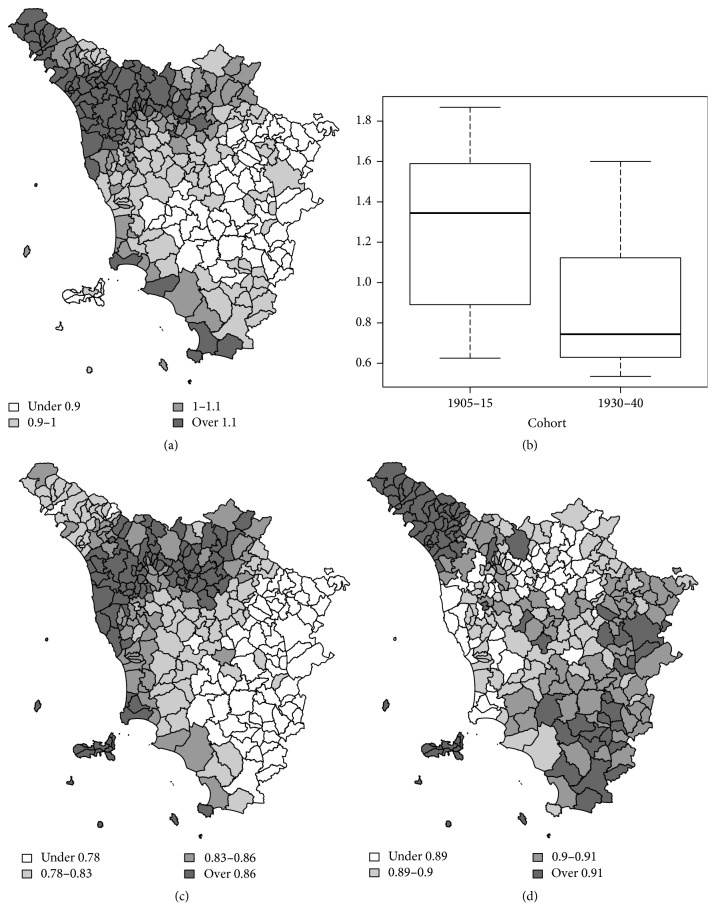
Results from SMBC model: the common clustering term exp⁡(*ψ*_*i*_^(*s*)^) (a), the distribution of the *ω*_1_ and *ω*_2_ parameters (b), the distribution and the specific clustering terms exp⁡(*ψ*_*i*1_^(*p*)^) for birth-cohort 1905–15 (c), and exp⁡(*ψ*_*i*2_^(*p*)^) for birth-cohort 1930–40 (d).

The birth-cohorts 1905–15 and 1930–40 are also compared through the more parsimonious SMBC.  Figure 3 shows relative risks from SMBC for the 1905–15 and 1930–40 birth-cohorts.  Figure 4(a) shows the estimates of the exponentialized parameters *ψ*_*i*_^(*s*)^, that is, the common spatial distribution of relative risk for lung cancer for males in Tuscany (Italy) for 1905–15 and 1930-40. There is a high-risk area in the north-western part of the region and a low-risk area near the south-eastern border. This pattern has a strong connection with the spatial distribution of socioeconomic characteristics over the region. Indeed, the heavily industrialized and developed areas within Tuscany are located in the river Arno valley, from the capital, Florence, to Pisa and the main port of Livorno. At the beginning of 1900, the first industrial settlements were mainly close to the mountain areas. In the map, this pattern corresponds to the north-western part of the region.

Figure 4(b) also shows the estimates of the parameters *ω*_*t*_, namely, the relative importance of the common pattern for the two birth-cohorts 1905–15 and 1930–40. The box-plots suggest that the epidemic of lung cancer clearly declines.

Figures 4(c) and 4(d) illustrate the estimates of the exponentialized parameters *ψ*_*it*_^(*p*)^, that is, the specific spatial distribution of relative risk for birth-cohorts 1905–15 and 1930–40. For the birth-cohort 1905–15, there are some high values for the cities of Florence, Siena, Pisa, Livorno, Grosseto and for the industrial municipalities of Massa, Carrara, and Piombino. On the contrary, there is a prominent pattern for the 1930–35 birth-cohort, with higher relative risks for the municipalities of Mount Amiata (southern Tuscany). Furthermore, a decrease in the risk is observed for the cities (with the exception of Arezzo that moves in the opposite direction), mainly for Florence and its metropolitan municipalities (Scandicci, Signa, and Lastra a Signa). High risks are also observed in the municipality of Colle Val D'Elsa.

Consequently, an opposite trend can be observed in Arezzo, Colle Val D'Elsa, and the Mount Amiata areas. Results from the two different models are comparable. The proposed model seems to highlight more directly and parsimoniously the differences between two and more cohorts. The maps for the specific components are not oversmoothed, since there is minor information from the data but also a less structured model.

After the Second World War, the mines of Mount Amiata were supplying 50% of the world production of Cinnabar (a mineral consisting of mercury) and they remained the top supplier for decades. Cinnabar, with its vermilion red color, was used mainly as a primary colorant of walls, fabrics, and crockery. Before the advent of mining on a large scale, the economy of almost all the municipalities in the territory of Mount Amiata was based on agro-forestry-pastoral activities. The arrival of the mines leads to a striking transformation of this type of economy. The mining centers of Abbadia San Salvatore, Castellazzara, Piancastagnaio, and Santa Fiora reported a marked improvement in living standards. But the two occupational diseases typical of mining and metallurgical activities linked to mercury were silicosis and mercury-poisoning. Silicosis is a pneumoconiosis caused by prolonged inhaling (20–30 years) of dust containing small crystalline free silica particles. In the seventies, the mercury crisis hit the mines of Mount Amiata. In any case, the 1930–40 cohort of workers had been exposed for a long period from the postwar era when the mine was “in the fullest activity” to the closure of the mines in 1970, that is, 20–30 years of exposure. Moreover, exposure to crystalline free silica also involved the workers in the gold casting sector of Valtiberina and Arezzo and the glassware sector of Colle Val D'Elsa.

The units most at risk include those working in the metal mines, ceramics and glass factories, foundries, and the sandstone mining and granite industries. Workers with high dust exposure who develop silicosis are at increased risk of lung cancer; however, high levels of exposure to silica dust on its own are significant in the pathogenesis of lung cancer and silicosis is coincidental.

## 5. Discussion and Conclusions

In this paper, the space-time epidemic of lung cancer in Tuscany (Italy) is investigated. The analysis is performed via a hierarchical Bayesian model with shared and cohort-specific risk components.

It should be mentioned that this work is mainly a descriptive epidemiological investigation. In addition, three possible criticisms are the ecological bias, the misleading specification of the deprivation measure, and considering the lag of association between deprivation and mortality of 10 years. Despite such (and possibly other) limitations, our analysis yields some information on the space-time behavior of lung cancer in Tuscany and gives a few hints on the etiology of this disease.

It should be also noted that socioeconomic factors, when used at aggregate level, could give rise to the problem of “ecological fallacy.” However, they allow for identifying homogeneous groups, to study large populations at low costs, and to address questions of environmental health that might be difficult or impossible to study using other approaches (see [42], for a review of ecological analysis).

Lung cancer mortality for males in Tuscany has increased over the last 40-year period (1971–2010), showing a strong epidemic curve with the highest rates for the birth-cohorts born between 1920 and 1930. The geographical distribution of relative risk is very spatially structured and the north-western areas have a higher risk (see also [43]) due to the location of industrial plants [44]. The resulting pattern has an interesting interpretation as it highlights small towns with industrial plants in the north-west of Tuscany, the Massa-Carrara, Piombino, and the Island of Elba (with coke and iron industries since 1905). The spatial patterns suggest a role played by occupational and environmental factors. For males, the specific high-risk municipalities correspond to early industrialized areas. Lung cancer epidemics are now decreasing in all the municipalities of Tuscany. The overall time trend is moving toward greater homogeneity among areas. However, some areas (the municipalities of Mount Amiata, Arezzo, and Colle Val D'Elsa) show the opposite behavior for the younger cohorts: a new occupational risk factor, namely, silica, could be the cause of lung cancer in workers in mines and the gold and glass sectors.

The role of silicosis in the development of lung cancer, associated with silica exposure, remains controversial. Some studies indicate an increased risk of lung cancer from exposure to silica, and others limit such association to individuals with silicosis, while others show no association at all. The association has been studied for many decades. See Lynge et al. [45], Hnizdo and Sluis-Cremer 1991 [46], Amandus and Costello [47], NIOS [48], EC 2006 [49], Maciejewska [50], Vida et al. [51], MH [52], Chen et al. [53], and Liu et al. 2013 [54]. Although controversial, the decision taken in 1997 by the International Agency for Research on Cancer (IARC) [55] classified the respirable fraction of crystalline silica as a carcinogen of the first group for humans. This was followed by several epidemiological studies in different industrial fields, meta-analyses and reviews. However, unequivocal conclusions were not always reached. In 2002, the Italian Silica Network (consisting of the regions, provinces, INAIL, ISPESL, National Institute of Health, local health authorities, and scientific research centers) was set up in the aim of intervening in the field of prevention which lacked the required completeness and clarity of legislation at a European level. An international estimation conducted in 2003 by CAREX (CARcinogen EXposure, Canada) suggested that in Italy more than 254 thousand people were exposed to word-related crystalline silica risks, 12–15 thousand of whom in Tuscany. In 2004, the Region of Tuscany launched a project aimed at the prevention, measurement, and intervention in critical sectors such as quarries, construction sites, glass factories, and cement plants. Silicosis, the terrible plague which in the past, and only until a few decades ago, accompanied the industrial and economic development of our country, is not almost completely eradicated. However, still far from being won, in Italy as in the rest of Europe, is the battle against another big risk that those who work with crystalline free silica are exposed to lung cancer.

## Figures and Tables

**Table 1:** Incidence rates (×100,000) and number of death cases (in parentheses). Lung cancer for males in Tuscany (Italy), 1971–2010, for age-class, calendar period, and birth-cohort.

Age-class	Birth-cohort	Calendar period							
		1971–74	1975–79	1980–84	1985–89	1990–94	1995–99	2000–04	2005–10
	Birth-cohort	1925–35	**1930**–**40**	1935–45	1940–50	1945–55	1950–60	1955–65	1960–65
40–44	1925–35	21.611 (106)	**22.764 (135)**	19.425 (116)	19.750 (117)	14.356 (86)	8.241 (48)	7.919 (51)	4.446 (40)
45–49	1920–30	43.225 (218)	44.661 (273)	**47.661 (278)**	46.078 (273)	32.182 (190)	26.567 (161)	23.154 (134)	16.185 (130)
50–54	1915–25	75.662 (342)	91.121 (568)	108.292 (642)	**85.201 (489)**	76.188 (446)	68.585 (398)	54.442 (324)	36.807 (261)
55–59	1910–20	128.517 (467)	150.719 (780)	187.735 (1112)	180.141 (1031)	**143.195 (802)**	113.619 (650)	100.928 (569)	78.371 (554)
60–64	**1905**–**15**	**190.339 (816)**	229.643 (1030)	247.445 (1225)	292.444 (1633)	259.192 (1413)	**201.356 (1081)**	174.847 (967)	162.399 (1089)
65–69	1900–10	279.517 (960)	**319.244 (1500)**	370.082 (1445)	352.777 (1548)	394.758 (2018)	345.721 (1731)	**287.057 (1429)**	244.304 (1525)
70–74	1895–05	286.059 (714)	385.879 (1324)	**432.871 (1672)**	468.204 (1562)	456.730 (1740)	478.094 (2123)	420.203 (1861)	**354.047 (1947)**
75–79	1890–00	264.585 (385)	344.589 (743)	443.843 (1092)	**495.515 (1449)**	521.901 (1378)	518.007 (1537)	525.898 (1889)	492.426 (2200)
80–84	1885–95	145.496 (128)	270.123 (293)	407.835 (517)	489.365 (756)	**487.923 (971)**	532.960 (963)	556.287 (1170)	579.774 (1834)
85+	1880–90	129.855 (57)	153.766 (92)	257.543 (165)	345.532 (269)	365.507 (385)	**445.953 (619)**	466.355 (683)	543.954 (1137)
